# Elastase-2, a Tissue Alternative Pathway for Angiotensin II Generation, Plays a Role in Circulatory Sympathovagal Balance in Mice

**DOI:** 10.3389/fphys.2017.00170

**Published:** 2017-03-23

**Authors:** Christiane Becari, Marina T. Durand, Alessander O. Guimaraes, Renata M. Lataro, Cibele M. Prado, Mauro de Oliveira, Sarai C. O. Candido, Paloma Pais, Mauricio S. Ribeiro, Michael Bader, Joao B. Pesquero, Maria C. O. Salgado, Helio C. Salgado

**Affiliations:** ^1^Department of Physiology, Ribeirão Preto Medical School, University of São PauloRibeirão Preto, Brazil; ^2^Department of Pharmacology, Ribeirão Preto Medical School, University of São PauloRibeirão Preto, Brazil; ^3^Department of Cardiovascular Diseases, Mayo ClinicRochester, MN, USA; ^4^Department of Medicine, University of Ribeirão PretoRibeirão Preto, Brazil; ^5^Max Delbruck Center for Molecular MedicineBerlin, Germany; ^6^Department of Biophysics, Federal University of São PauloSão Paulo, Brazil; ^7^Department of Pathology, Ribeirão Preto Medical School, University of São PauloRibeirão Preto, Brazil; ^8^Department of Surgery and Anatomy, Ribeirão Preto Medical School, University of São PauloRibeirão Preto, Brazil; ^9^Berlin Institute of Health, Charité—University Medicine BerlinBerlin, Germany; ^10^German Center for Cardiovascular Research, Partner Site BerlinBerlin, Germany

**Keywords:** elastase-2, angiotensina II, sympathovagal balance, analysis spectral, knockout mice

## Abstract

*In vitro* and *ex vivo* experiments indicate that elastase-2 (ELA-2), a chymotrypsin-serine protease elastase family member 2A, is an alternative pathway for angiotensin II (Ang II) generation. However, the role played by ELA-2 *in vivo* is unclear. We examined ELA-2 knockout (ELA-2KO) mice compared to wild-type (WT) mice and determined whether ELA-2 played a role in hemodynamics [arterial pressure (AP) and heart rate (HR)], cardiocirculatory sympathovagal balance and baroreflex sensitivity. The variability of systolic arterial pressure (SAP) and pulse interval (PI) for evaluating autonomic modulation was examined for time and frequency domains (spectral analysis), whereas a symbolic analysis was also used to evaluate PI variability. In addition, baroreflex sensitivity was examined using the sequence method. Cardiac function was evaluated echocardiographically under anesthesia. The AP was normal whereas the HR was reduced in ELA-2KO mice (425 ± 17 vs. 512 ± 13 bpm from WT). SAP variability and baroreflex sensitivity were similar in both strains. The LF power from the PI spectrum (33.6 ± 5 vs. 51.8 ± 4.8 nu from WT) and the LF/HF ratio (0.60 ± 0.1 vs. 1.45 ± 0.3 from WT) were reduced, whereas the HF power was increased (66.4 ± 5 vs. 48.2 ± 4.8 nu from WT) in ELA-2KO mice, indicating a shift toward parasympathetic modulation of HR. Echocardiographic examination showed normal fractional shortening and an ejection fraction in ELA-2KO mice; however, the cardiac output, stroke volume, and ventricular size were reduced. These findings provide the first evidence that ELA-2 acts on the sympathovagal balance of the heart, as expressed by the reduced sympathetic modulation of HR in ELA-2KO mice.

## Introduction

The beneficial effects of angiotensin-converting enzyme (ACE) inhibitors in the treatment of arterial hypertension, congestive heart failure, and other cardiovascular diseases are well documented (Hansson et al., 1999; Jorde et al., 2000; Yusuf et al., 2000; Lindholm et al., 2002; Jandeleit-Dahm et al., 2005; Feldstein, 2014; Te Riet et al., 2015). However, patients chronically treated with appropriate doses of ACE inhibitors do not show permanent attenuation of angiotensin II (Ang II) plasma levels (Jorde et al., 2000; Farquharson and Struthers, 2002), indicating the existence of an ACE-independent alternative pathway for Ang II generation. Elastase-2 (ELA-2), a chymotrypsin-serine protease elastase family member 2A, is an alternative Ang II generator. This enzyme is widely distributed in several murine organs, such as the lung, pancreas, liver, heart, kidney, and blood vessels (Santos et al., 2002; Becari et al., 2011).

The functional role of ELA-2 for Ang II generation was demonstrated in both *in vitro* and *ex vivo* mesenteric arterial beds (Santos et al., 2002, 2003), hearts and carotid arteries (Becari et al., 2005, 2011). However, the *in vivo* role of ELA-2 remains unclear, and there are no reports providing support for its participation in cardiocirculatory regulation under physiological conditions. Thus, to investigate whether ELA -2 plays a role in cardiocirculatory regulation, our laboratory developed a knock-out mouse strain for the ELA-2 gene (ELA-2KO).

This study hypothesized that ELA-2 may have a significant contribution in cardiocirculatory regulation due to its wide tissue distribution. Therefore, knock out mice were used to examine changes in cardiovascular parameters (arterial pressure and heart rate) and sympathovagal balance that may be associated with the lack of ELA-2. To test this hypothesis, the cardiocirculatory sympathovagal balance was examined in the time and frequency domains and the baroreflex function, assessed by baroreflex sensitivity, was investigated in conscious ELA-2KO and wild-type mice (Bertinieri et al., 1985; Di Rienzo et al., 2001). Moreover, a nonlinear method for investigating heart rate variability (HRV), based on symbolic analysis, was also applied (Guzzetti et al., 2005a; Porta et al., 2007). Additionally, cardiac function was evaluated using echocardiography to assess the cardiac output, stroke volume, fractional shortening, ejection fraction, and ventricular dimension in anesthetized ELA-2KO mice. Cardiac morphological aspects, such as myocyte size, cardiac index, ventricular area, ventricular wall thickness, and collagen density were also studied histologically.

Given the potential for future research in this field, the aim of this study was to investigate the role of tissue ELA2 in the control of hemodynamics and cardiac function by characterizing cardiocirculatory sympathovagal balance, baroreflex sensitivity, and cardiac physiological parameters in ELA-2KO mice.

## Methods

### Ethical approved

All studies involving animals are reported in accordance with the ARRIVE guidelines (Kilkenny et al., 2010; McGrath et al., 2010, 2015). All procedures were approved by the Ethics Committee in Animal Research of the Ribeirão Preto Medical School (University of São Paulo, Ribeirão Preto, SP, Brazil; Protocol # 058/2012).

### Animals

Experiments were performed using wild-type (WT) male mice (C57Bl/6J) and ELA-2 KO mice (Cela2a^Tm1Bdr^). Their age ranged from 10 to 15 weeks and weighed from 25 to 32 g. ELA-2KO and WT mice were supplied, respectively, by Dr. Helio C. Salgado's Laboratory and the Animal Facility of the University of Sao Paulo in Ribeirao Preto (SP, Brazil). Mice were fed standard chow diet and tap water *ad libitum* and were housed under controlled temperature (22°C) and 12-h dark-light cycles.

### ELA-2 knockout homozygous mice

ELA-2KO (B6;129P2-Cela2a^Tm1Bdr^) mice were developed as described in the Supplemental Data.

### Genotyping

Tail tissue genomic DNA was obtained and was used to amplify the target gene by polymerase chain reaction (PCR). The expected amplicon size for the knockout allele amplified with the primers ElaF (5′AGAAACTATGTCTGCTATGTCAC3′) and pELAloxr2 (5′TTCTTGAACTGATGGCGAGC3′) is 295 bp, and the WT allele should yield an amplicon of 345 bp using the primers ElaF and Ela WTR (5′TTTACAGATGAGGAAGTCACC3′).

#### Echocardiography

The mice were lightly anesthetized with 1.5% isoflurane in 100% O_2_. The chest wall was shaved, and a small gel standoff was placed between the chest and a 30-MHz scan head interfaced with a Vevo 2100 High-Resolution Imaging System (VisualSonics, Toronto, ON, Canada). The body temperature was monitored during echocardiography. High-resolution B mode and M mode images were acquired with a 30-MHz transducer. Continuous, standard electrocardiography was recorded using electrodes placed on the animal's extremities. Systolic measurements were recorded at the point of minimal cavity dimension using the leading edge method of the American Society of Echocardiography. Ejection fraction and fractional shortening were calculated and used as the determinant of left ventricle systolic function.

### Catheter implantation

Mice were anesthetized with isoflurane (5% for induction and 2–3% for maintenance) provided by an isoflurane vaporizer (Calibrated Vaporizer Takaoka, model ISOVAPOR 1224, K. Takaoka Ind. Com. Ltda., São Paulo, Brazil). An arterial catheter composed of Micro-Renathane tubing (0.64-mm outer diameter, 0.30-mm inner diameter; Braintree Scientific, Braintree, MA, USA) was chronically implanted into the femoral artery for arterial pressure measurement. The catheter was filled with heparinized saline (100 i.u. ml^−1^), capped, tunneled subcutaneously and exposed on the back of the neck. Mice received penicillin/streptomycin (200 i.u. g^−1^ and 80 μg g^−1^, respectively) and were allowed to recover for 4 days.

### Arterial pressure and heart rate

Experiments were performed in conscious unrestrained mice, in their own cages, 4 days after surgery. On the day of the experiment, the arterial catheter was connected to a pressure transducer (model DPT-100 Deltran®; Utah Medical Products, Midvale, UT, USA) and mice were allowed to rest for 30–45 min. The basal arterial pressure and heart rate signals were recorded continuously for 30 min to evaluate the arterial pressure, pulse interval variability and baroreflex sensitivity. Data acquisition was performed using the Dataquest ART™ System software (Data Sciences International, St. Paul, MN) at a sample frequency of 2 kHz. All experiments were performed during the daytime to avoid any interference from circadian rhythms.

Pulsatile arterial pressure was analyzed using specific software that detects inflection points in periodic waves (LabChart version 7.0; AD Instruments, Sydney, Australia). A beat-by-beat time series of systolic arterial pressure (SAP) was generated. A series of pulse intervals was obtained by measuring the intervals between consecutive arterial pressure values, which were used to measure HRV.

#### Arterial pressure and pulse interval variability

The baseline SAP and pulse interval were used for variability analysis using customized software (CardioSeries-version 2.4, http://sites.google.com/site/cardioseries). For measures on the time domain, the standard deviation (SD) of successive normal values of the pulse interval (SDNN) and square root of the mean of the sum of the square of differences between adjacent pulse intervals (RMSSD) were considered. The overall variability of the arterial pressure was also calculated using the SD of the SAP series. The arterial pressure and pulse interval variability were also studied in the frequency domain by spectral analysis. The time series of the SAP and pulse interval were resampled to 12 Hz by cubic spline interpolation and were divided into contiguous segments of 512 values, which overlapped by 50%. Using a Hanning window, the spectrum of each segment of either the SAP or pulse interval series was calculated using the fast Fourier transform method and was integrated into two frequency bands: low frequency (LF; 0.1–1 Hz) and high frequency (HF; 1–5 Hz). The results are expressed in absolute units (ms^2^ or mmHg^2^) and normalized units (nu). The HRV was used to investigate the relative cardiac autonomic modulation from the sympathetic (LF power) and parasympathetic (HF power) systems in humans and rodents. The ratio of LF to HF (LF/HF) was used to represent the sympathovagal balance (Thireau et al., 2008; Thayer et al., 2010).

#### Symbolic analysis

To distinguish between sympathetic and parasympathetic cardiac modulation, symbolic analysis of three-beat sequences was performed using customized software (CardioSeries version 2.4, http://sites.google.com/site/cardioseries) to detect changes in autonomic modulation (Guzzetti et al., 2005b; Porta et al., 2007). Pulse interval sequences of ~60 s were selected from continuous basal recordings. The full range of sequences was uniformly spread across six levels (from 0 to 5), transforming them into a sequence of integers (i.e., symbols). Patterns, represented by sequences of three symbols, were constructed based on the sequence of symbols, and all possible patterns were divided into the following four groups (Guzzetti et al., 2005b):

1. Patterns with no variations (0 V; three symbols equal, indicating sympathetic modulation);
2. Patterns with one variation (1 V; two consequent symbols equal and the remaining symbol was different, indicating both sympathetic and parasympathetic modulation);
3. Patterns with two similar variations (2 LV; three symbols forming an ascending or a descending ramp, indicating parasympathetic modulation);
4. Patterns with two dissimilar variations (2 UV; the three symbols forming a peak or valley, indicating parasympathetic modulation);

The rates of occurrence of these patterns (0 V, 1 V, 2 LV, and 2 UV) were quantified. Approximately 12 series of pulse intervals of 60 s each were used for each animal.

#### Spontaneous baroreflex sensitivity

Spontaneous baroreflex sensitivity was assessed using the sequence technique described by Bertinieri et al. (1985). Over a 60-min selected period, a beat-by-beat time series of SAP and pulse intervals were scanned. The objective of the method was to search for sequences of at least four consecutive beats in which increases in arterial pressure were followed by pulse interval lengthening (up sequence) and decreases in arterial pressure were followed by pulse interval shortening (down sequence). The slope of the linear regression line between the SAP and pulse interval was taken as the measure of spontaneous baroreflex sensitivity. The baroreflex effectiveness index (BEI), which is complementary information to the spontaneous baroreflex sensitivity, was also calculated (Di Rienzo et al., 2001). The BEI is defined as the ratio between the number of SAP ramps followed by reflex pulse interval ramps and the total number of SAP ramps, either accompanied by the corresponding reflex pulse interval ramps or observed over a given time frame (Di Rienzo et al., 2001).

### Morphological analysis

After the hemodynamic recordings, the animals were anesthetized with ketamine and xylazine (1:1; Ketamina Agener União Saúde Animal, Embu-Guaçu, SP, Brazil; Dopaser Hertape Calier Saúde Animal S/A, Juatuba, MG, Brazil). Then, their entire hearts were harvested, rinsed using 10% KCl solution to stop them in diastole, and they were then weighed. The hearts were cut transversely and fixed in phosphate-buffered 10% formalin for 24–48 h. For histological processing, the specimens were embedded in paraffin and serially cut in 6-μm thick slices. The sections were subsequently stained with picrosirius red or hematoxylin and eosin. The analyses were performed as described elsewhere in Durand et al. (2014) and Lataro et al. (2013). Briefly, the heart picrosirius red-stained sections were used to estimate the volume fraction (%) of collagen, whereas 15 microscopic fields were randomly chosen and the mean value was subsequently calculated. The minor diameter of the myocytes of the left ventricle was measured in hearts stained with hematoxylin and eosin, whereas approximately 30 microscopic fields were randomly chosen and the mean value was calculated. Since the myocardial fiber has a tubular shape, the smallest axis is a measure perpendicular to the nucleus, no matter the myocardial fiber is sectioned in longitudinal, oblique, or transversal manner. These measurements were obtained from the nuclear region of those cardiomyocytes where the nucleus was centrally positioned. The sections were analyzed using the video microscopy software Leica Qwin (Leica Imaging Systems, Cambridge, UK) and the public-domain software NIH ImageJ (developed by U.S. National Institutes of Health and available at). Cardiac index (mg/g) was calculated by ratio heart weight (mg) on body weight (g).

### Group sizes and blinding

Experiments were carried out in 5 independent experiments from WT and ELA-2 KO mice. In both groups it was also measured the arterial pressure and heart rate combined with the evaluation of hemodynamic (arterial pressure and pulse interval) variability, symbolic analysis, spontaneous baroreflex sensitivity and morphological analysis. While one investigator performed echocardiographic experiments, another researcher blinded for the experimental groups performed the analyses. The hearts were collected after stopping in diastole and were kept in KCL (10%). Therefore, the cardiac wall thickness was measured in diastole.

### Statistical analysis

Statistical analyses were performed using unpaired Student's *t-*test. Values are expressed as the mean ± SEM. Differences were considered significant at *P* < 0.05. All statistical analyses were performed using GraphPad Prism Software.

## Results

### Heart rate and arterial pressure variability

**Figure 1:**
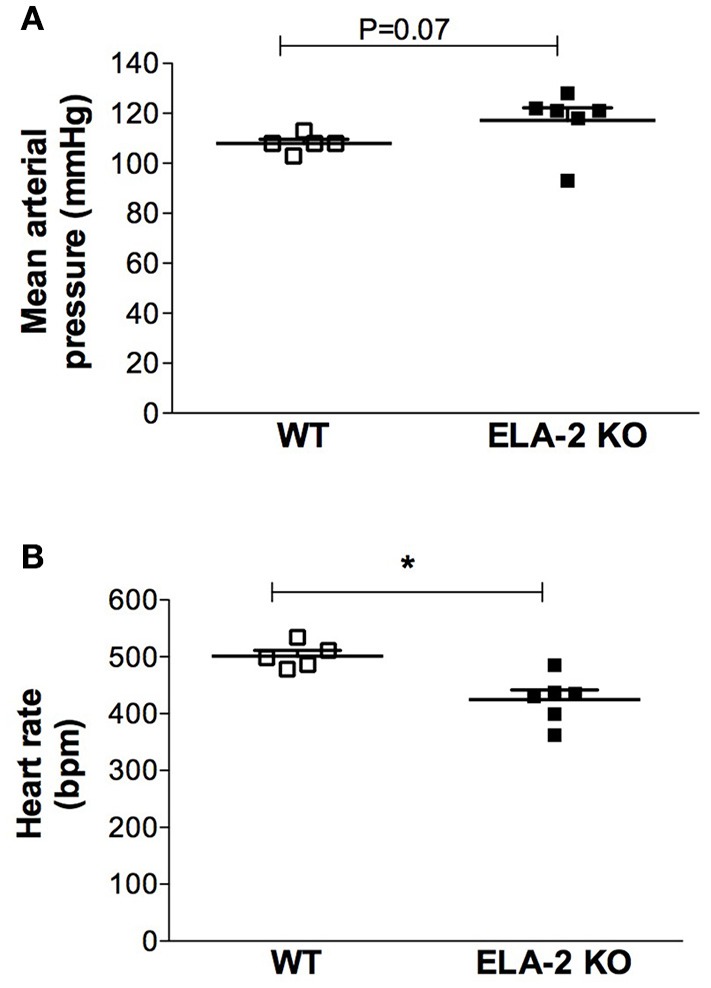
**Basal mean arterial pressure (A)** and heart rate **(B)** from WT (*n* = 5) and ELA-2KO (*n* = 6) mice. WT, wild C57Bl mice; ELA-2KO, elastase-2 knockout mice. Values are presented as the mean ± SEM. ^*^p < 0.05 compared with WT.

**Figure 2:**
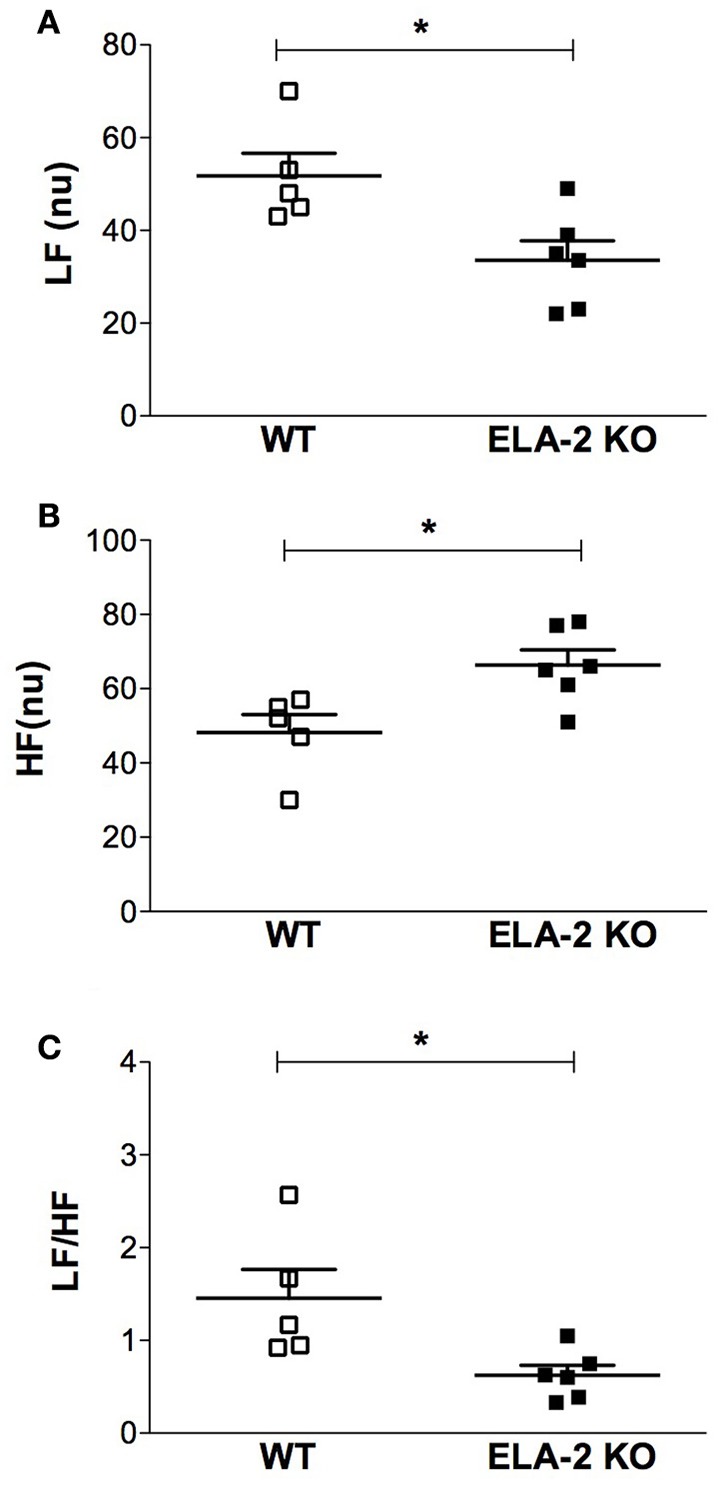
**Spectral power density of heart rate, in normalized units (nu), for (A)** low-frequency (LF) and **(B)** high-frequency (HF) bands, and **(C)** LF/HF from WT (*n* = 5) and ELA-2KO (*n* = 6) mice. WT, wild C57Bl mice; ELA-2KO, elastase-2 knockout mice. Values are presented as the mean ± SEM. ^*^p < 0.05 compared to WT.

**Figure 3:**
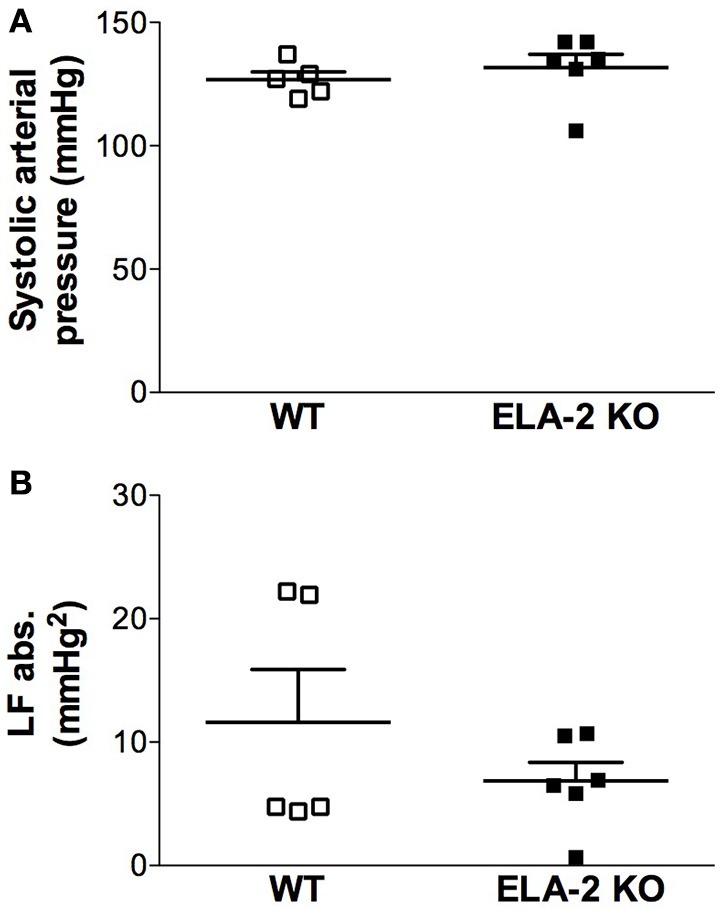
**Systolic arterial pressure (A)** and spectral power density of systolic arterial **(B)** pressure, in absolute units (abs), for low-frequency (LF) bands from WT (*n* = 5) and ELA-2KO (*n* = 6) mice. WT, wild-type C57Bl mice; ELA-2KO, elastase-2 knockout mice. Values are presented as the mean ± SEM.

ELA-2KO mice showed a lower basal heart rate compared to WT mice (Figure 1B), whereas there was no difference in the basal mean arterial pressure (Figure 1A). Data from cardiovascular variability analyses are shown in Table 1. ELA-2KO mice showed reduced power (nu) for the LF band (Figure 2A), higher power (nu) for the HF band (Figure 2B) and a lower LF/HF ratio (Figure 2C). However, the SAP variability, SAP (Figure 3A) and LF band (Figure 3B) were not different in ELA-2KO mice compared to WT mice.

**Table 1:** **Pulse interval (PI) and systolic arterial pressure (SAP) variability from wild type (WT) and knockout mice for tissue enzyme elastase-2 (ELA-2KO)**.

	**WT**	**ELA-2 KO**	***p*-value**
**PULSE INTERVAL VARIABILITY**			
SDNN, ms	7.1 ± 1.3	5.8 ± 1.3	0.522
RMSSD, ms	7.4 ± 1.4	6.3 ± 1.4	0.593
VLF, ms	21.2 ± 5.6	12 ± 3.4	0.200
LF, ms^2^	22.6 ± 14.8	17.4 ± 9.6	0.724
HF, ms^2^	13.4 ± 3.5	23.1 ± 8.5	0.319
LF, nu	51.8 ± 4.8	33.6 ± 5.0	**0.032**
HF, nu	48.2 ± 4.8	66.4 ± 5.0	**0.032**
LF/HF	1.45 ± 0.3	0.6 ± 0.1	**0.039**
**SYSTOLIC ARTERIAL PRESSURE VARIABILITY**			
SDNN, mmHg	3.6 ± 0.3	3.5 ± 0.6	0.975
LF, mmHg^2^	11.6 ± 4.3	6.9 ± 1.8	0.343

*SDNN, standard deviation of successive normal values; RMSSD, square root of the mean of the sum of the square of differences between adjacent pulse intervals; LF, low frequency; HF, high frequency; nu, normalized units. Data are presented as mean , SEM, WT-n = 5, ELA-2 KO n = 6. Bold values means p < 0.05 compare with WT*.

**Figure 4:**
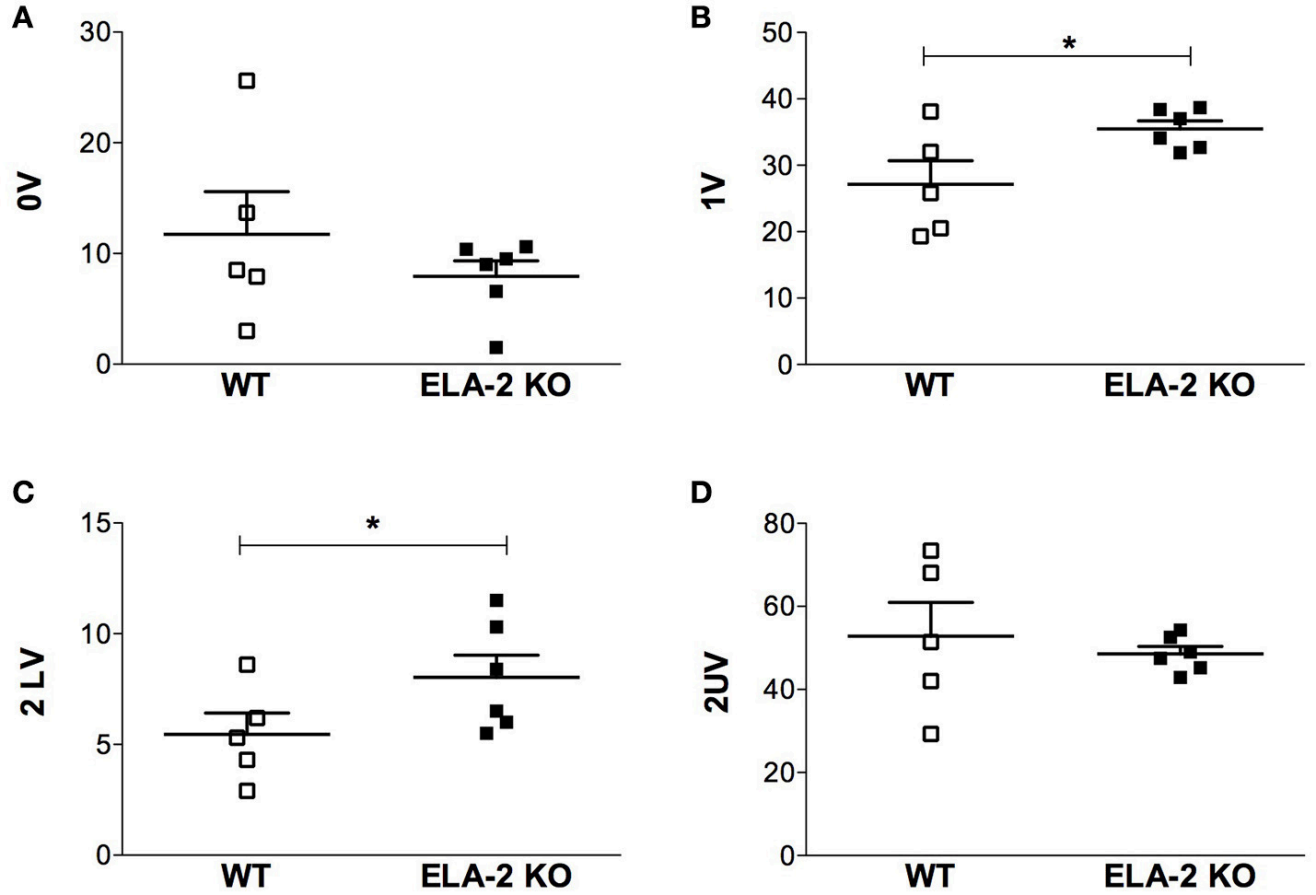
**Symbolic analysis from WT and ELA-2KO mice**. **(A)** 0V: three symbols equal indicate sympathetic modulation; **(B)** 1V: one variation, two symbols equal and one different, indicate both sympathetic and parasympathetic modulation; **(C)** 2LV: two like variations indicate parasympathetic modulation; **(D)** 2UV: two unlike variations indicate parasympathetic modulation. WT, wild C57Bl mice; ELA-2KO, elastase-2 knockout mice. Values are presented as the mean ± SEM. ^*^p < 0.05 compared to WT (*n* = 5–6).

Data from symbolic analysis, a tool that also evaluates sympathetic and parasympathetic cardiac modulation (Guzzetti et al., 2005b; Porta et al., 2007), are shown in Figure 4. The 0 V pattern occurrence, which indicates cardiac sympathetic modulation, was similar between ELA-2 KO and WT mice (Figure 4A). Furthermore, cardiac parasympathetic modulation, assessed by 2 LV pattern occurrence, was higher in ELA-2KO than WT mice (Figure 4C). The sympathetic and parasympathetic modulation assessed by 1 V pattern occurrence, was higher in ELA-2KO than WT mice (Figure 4B). Nevertheless, 2 UV pattern occurrence, which in healthy subjects seems to be under vagal control (Tobaldini et al., 2009), was similar between ELA-2KO and WT mice (Figure 4D).

### Spontaneous baroreflex sensitivity

**Figure 5:**
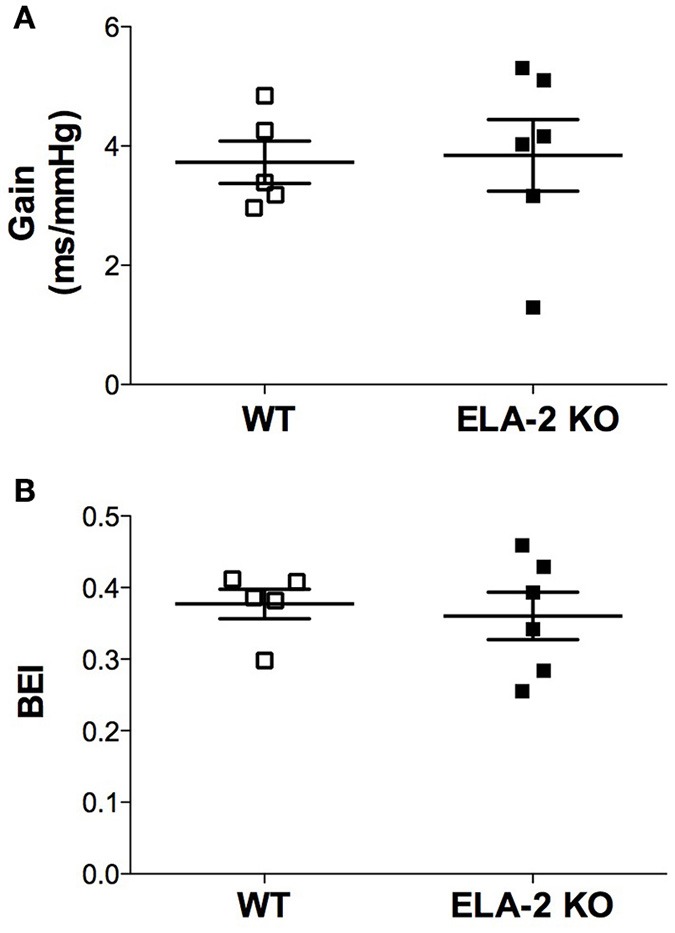
**Spontaneous baroreflex sensitivity: (A)** gain and **(B)** baroreflex effectiveness index (BEI) for WT (*n* = 5) and ELA-2KO (*n* = 6) mice. WT, wild C57Bl mice; ELA-2KO, elastase-2 knockout mice. Values are presented as the mean ± SEM.

ELA-2KO mice did not show alteration of the spontaneous baroreflex sensitivity indices, i.e., gain and BEI (Figures 5A,B).

### Cardiac function and remodeling

**Figure 6:**
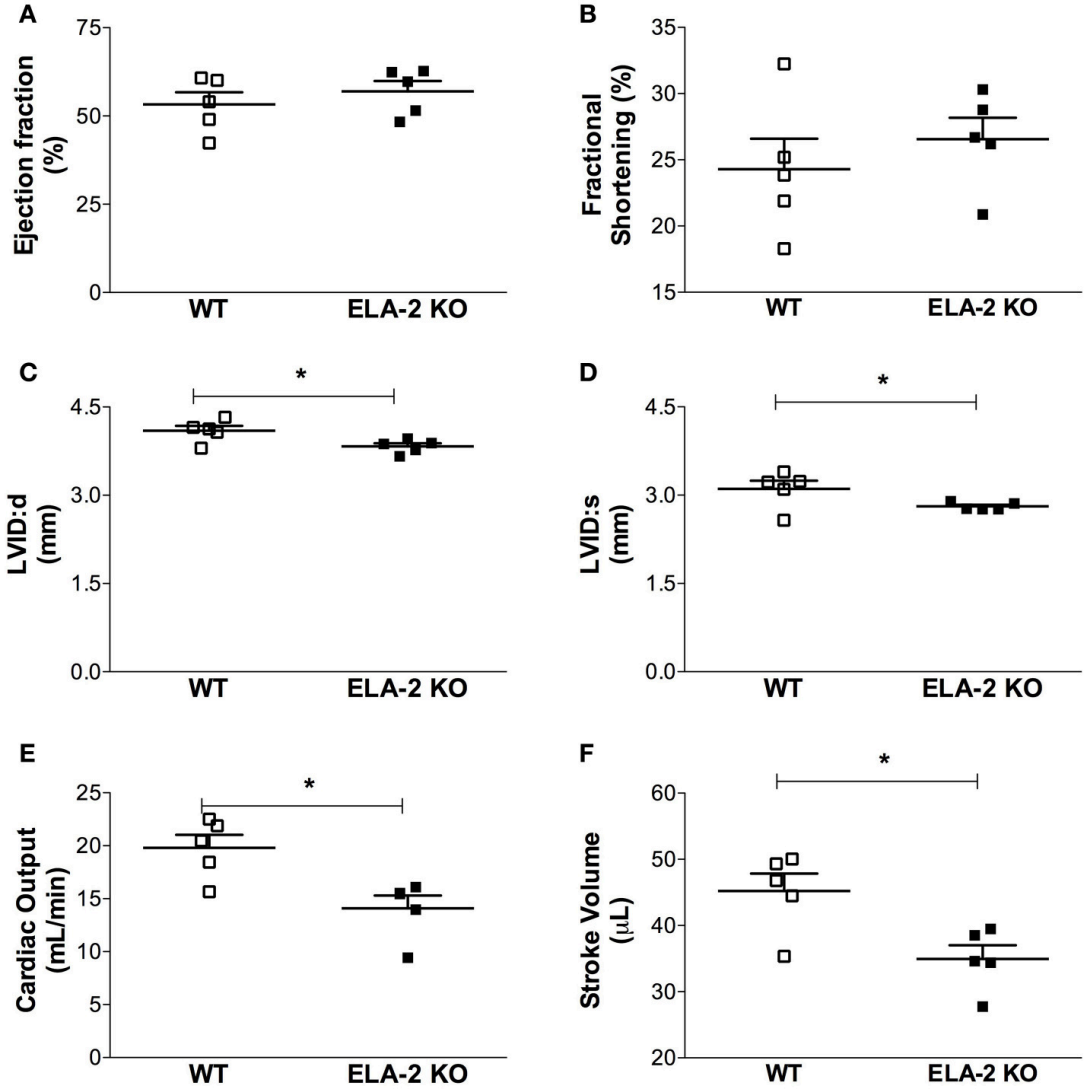
**Echocardiography from WT (***n*** = 5) and ELA-2 KO (***n*** = 5) mice**. Cardiac function assessment: **(A)** ejection fraction; **(B)** fractional shortening, **(C)** LVIDd (end diastolic left ventricular internal diameter); **(D)** LVIDs (end systolic left ventricular internal diameter); **(E)** cardiac output; **(F)** stroke volume. WT, wild C57Bl mice; ELA-2KO, elastase-2 knockout mice. Values are presented as the mean ± SEM. ^*^p < 0.05 compared to WT.

**Figure 7:**
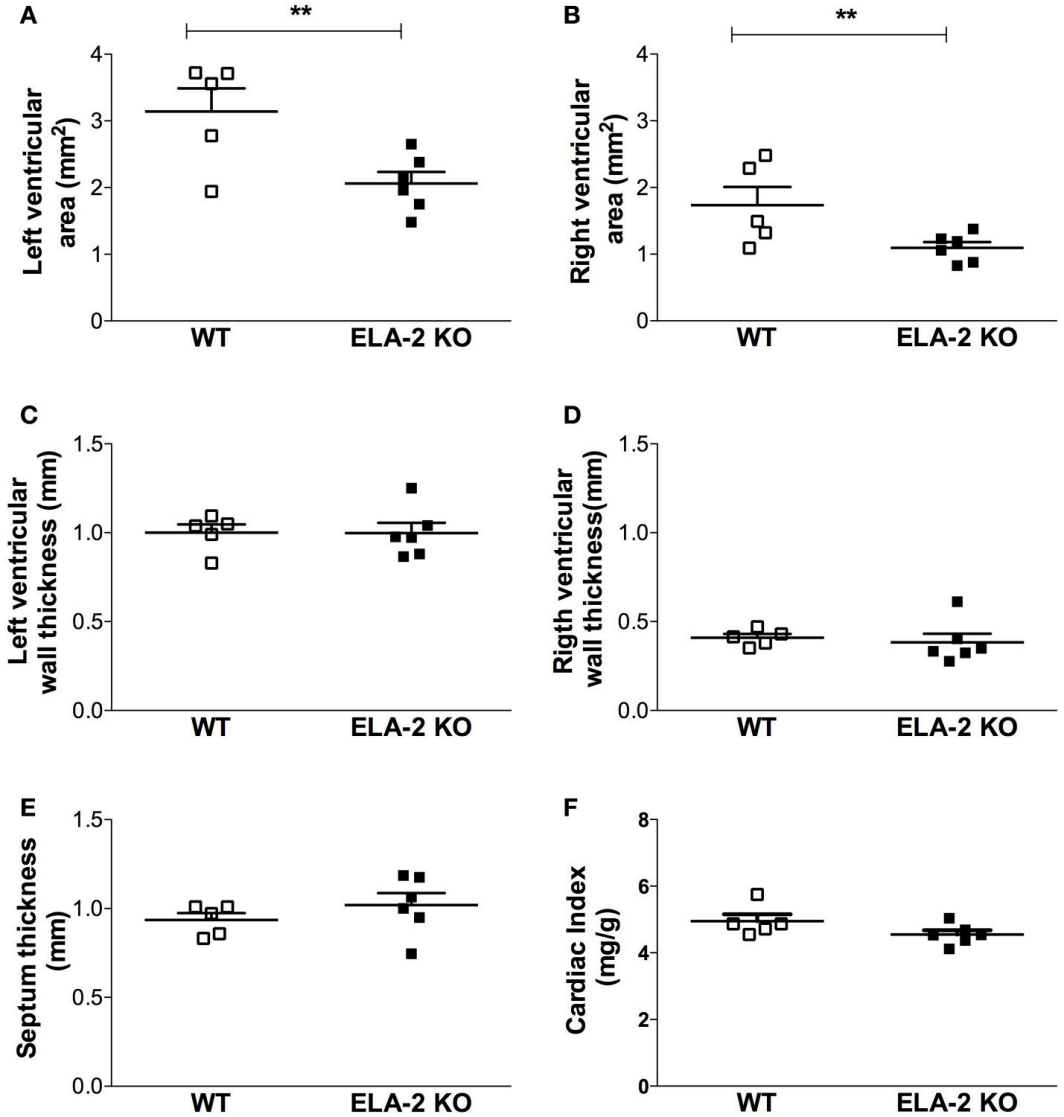
**Histological analysis of the left ventricle from WT (***n*** = 5) and KO ELA-2 (***n*** = 6) mice**. Bar graphs show the following: **(A)** left ventricular area; **(B)** right ventricular area; **(C)** left ventricular wall thickness; **(D)** right ventricular wall thickness; **(E)** septum thickness; **(F)** cardiac index. WT, wild C57Bl mice; ELA-2KO, elastase-2 knockout mice. Values are presented as the mean ± SEM. ^**^p < 0.01 compared to WT.

**Figure 8:**
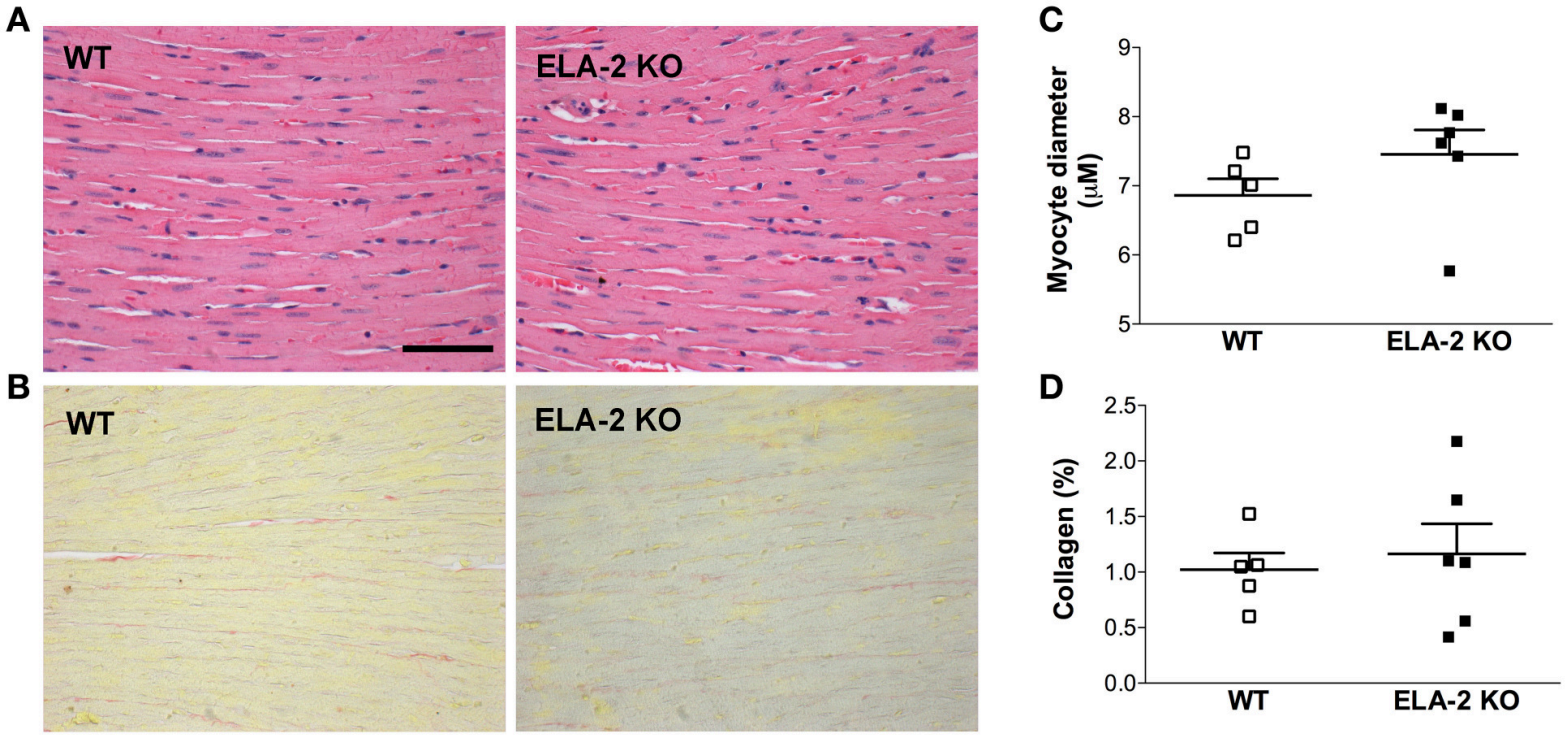
**Histological analysis of the left ventricle from WT (***n*** = 5) and KO ELA-2 (***n*** = 6) mice. (A):** hematoxylin and eosin staining illustrates the myocyte diameter (scale bar = 50 μm). **(B)**: picrosirius red staining illustrates the collagen density (scale bar = 50 μm). **(C)**: myocyte diameter from the left ventricle. **(D):** collagen fraction from the left ventricle. WT, wild C57Bl mice; ELA-2KO, elastase-2 knockout mice.

The assessment of cardiac function showed that the heart rate under anesthesia was reduced in ELA2KO mice compared to WT (388 ± 16 vs. 438 ± 4.8 bpm from WT). The assessment of cardiac function showed that the heart rate under anesthesia was reduced in ELA2KO mice compared to WT (388 ± 16 vs. 438 ± 4.8 bpm from WT, *p* = 0.025) mice, whereas the ejection fraction and fractional shortening were similar between ELA-2KO and WT mice (Figures 6A,B). For the left ventricle dimensions, ELA-2KO mice showed smaller diameters than WT mice (Figures 6C,D). Indeed, ELA-2KO mice showed smaller left ventricles than WT mice, despite similar heart (138.5 ± 8.2 vs. 129.9 ± 2.4 mg from WT) and body weights (29.1 ± 1.0 vs. 29.0 ± 0.5 g from WT). Cardiac output and stroke volume were lower in ELA-2KO than WT mice (Figures 6E,F). As shown by echocardiography, ELA-2KO mice showed significantly smaller left and right ventricle areas than WT mice (Figures 7A,B). Nonetheless, no differences in the left and right ventricle wall thickness were observed (Figures 7C,D). For the septum thickness and cardiac index, ELA-2KO mice did not show alterations compared to WT mice (Figures 7E,F). Furthermore, the myocyte size and collagen density were similar in ELA-2KO and WT mice (Figure 8). Histological analysis confirmed the morphological alteration of hearts from ELA-2KO mice.

## Discussion

This is the first study *in vivo* describing that ELA-2 KO mouse and ELA-2 acts on the sympathovagal balance of the heart of ELA-2KO mice. The important finding was the significant alteration in the cardiac sympathovagal balance, characterized by increased parasympathetic and reduced sympathetic modulation. The lower heart rate, cardiac output, stroke volume, and reduced left ventricle are also interesting findings that may be related to autonomic dysregulation or associated with overall systemic change. Nevertheless, these findings provide the first evidence that ELA-2 plays a role in the maintenance of basal hemodynamics, contributing to cardiovascular homeostasis.

ELA-2 belongs to the chymotrypsin-like elastase family, member 2A. Ela-2 is considered the only representative of this family of proteases that is secreted outside the digestive tract and is involved in Ang II generation. ELA-2 is widely found in several organs such as lung, pancreas, liver, blood vessels (mesenteric, and carotid arteries), heart and kidney of rodents (Santos et al., 2003; Becari et al., 2005, 2011). The importance of this functional alternative pathway for Ang II generation was demonstrated in heart, carotid and mesenteric arteries from normotensive and spontaneously hypertensive (SHR) rats (Santos et al., 2002, 2003; Becari et al., 2005, 2011).

ELA-2KO mice presented a reduced heart rate with normal systemic arterial pressure. The ELA-2 deficiency could possibly decrease the cardiac inhibitory effect of Ang II on parasympathetic function, justifying the observed relative bradycardia in ELA-2KO mice. Ang II can enhance the sympathetic tone and facilitate catecholamine release from cardiac sympathetic nerve endings via AT_1_R (Reid, 1992; Brasch et al., 1993; Bezerra et al., 2001). By contrast, Takata et al. (2004) demonstrated that inhibition of ACE with captopril enhanced vagal nerve stimulation-evoked bradycardia in pithed rats, without affecting cardiac sympathetic neurotransmission. Furthermore, ELA-2 is a serine proteinase with broad substrate specificity; therefore, it may play important role on different systems other than the renin angiotensin system (RAS). Because the autonomic and morphological changes in ELA-2 KO mice are qualitatively and quantitatively different when RAS is simply inhibited, the hypothesis that the inhibition of Ang II is not the main or, at least, the only factor responsible for the physiological changes seen in ELA-2 KO mice is supported.

The main morphological finding shown by echocardiography was an apparent hypoplasic left ventricle in ELA-2KO mice combined with reduced stroke volume and cardiac output, despite no changes in body and heart weights or cardiac index. These morphological findings were confirmed histologically by significant reduction of the ventricles. Interestingly, there was no alteration of ventricular and septal wall thickness, myocyte diameter and collagen concentration.

The reduced cardiac output may be explained by the association of bradycardia and low stroke volume. Furthermore, the basal arterial pressure demonstrated a trend to increase (*p* = 0.07), likely due to an increase in the peripheral resistance. Indeed, unpublished data from our group demonstrated increased vascular resistance in this mouse strain. We recently investigated the importance of assessing local Ang II generation in peripheral resistance arteries on myocardial infarction. The fact that Ang II generation is increased and ELA-2 is responsible for Ang II generation in mesenteric resistance arteries from mice submitted to myocardial infarction, these findings provide novel information for the role played by ELA-2 upon vascular resistance in this pathological condition (Becari et al., 2017).

Heart rate and arterial pressure variability are extensively used to assess autonomic modulation of the cardiovascular system under clinical (Huikuri and Stein, 2013; Stein, 2013; Wu et al., 2014) and experimental conditions (Mansier et al., 1996; Wickman et al., 1998; Sabino et al., 2013). In the current study, the SAP variability in the time and frequency domains was similar between ELA-2KO and WT mice. However, the LF power of the pulse interval spectrum and LF/HF ratio were reduced, whereas the HF was increased in ELA-2KO mice, indicating a sympathovagal balance with a shift toward parasympathetic modulation of the heart. Clinical and experimental studies that evaluated heart rate variability after inhibition of ACE in cardiovascular disease also led to an increase in HF oscillations and decrease in both LF oscillations and the LF/HF ratio (Menezes et al., 2004; Albarwani et al., 2013; Chompoosan et al., 2014). These data indicate that the prevailing sympathetic drive observed in cardiovascular diseases can be blunted by ACE inhibition and its consequent reduction of Ang II availability. The results from symbolic analysis demonstrated an increase in 2 LV pattern occurrence, with no changes in 0 V and 2 UV in ELA2KO mice. Notably, according to studies that used these nonlinear normalized indices, the 1 V pattern occurrence represents parasympathetic and sympathetic modulation, whereas the 2 UV pattern occurrence represents only parasympathetic modulation of the heart (Porta et al., 2007; Durand et al., 2015). Collectively, the data from spectral and symbolic analysis suggest that ELA2KO mice have a shift toward parasympathetic over sympathetic modulation of the heart, which likely contributed to the basal bradycardia exhibited by these mice.

An approach for estimating spontaneous baroreflex sensitivity is a dynamic evaluation called the sequence technique (Bertinieri et al., 1985), and it has also been applied to conscious mice (Joaquim et al., 2004; Chen et al., 2005). Laude et al. (2008) showed that the spontaneous baroreflex sensitivity obtained through the sequence technique was highly correlated with spectral estimates and also demonstrated a vagal predominance on baroreflex control of heart rate in conscious mice. The results from our laboratory in conscious mice (Fazan et al., 2005) showed that baroreflex modulation of PI plays a role in the LF band, but not in the HF band of PI variability, mediated by both sympathetic and parasympathetic drives. Nevertheless, in the present study, a difference in baroreflex sensitivity was not observed between groups, suggesting that the increased parasympathetic modulation in ELA-2KO mice is not due to baroreflex sensitivity enhancement.

The deletion of ELA-2 in mice decreased basal heart rate and stroke volume, these observations suggest that ELA-2 plays a role in maintaining cardiac heart rate and contractility. Additionally, the reduced heart rate, cardiac output, stroke volume, and reduced left ventricle size are also interesting findings that might be related to the autonomic dysregulation, or associated with overall systemic change. These data indicate that ELA-2 may play a role on basal cardiac function and peripheral resistance. Increased peripheral resistance, a hallmark of chronic heart failure after myocardial infarction (Schrier and Abraham, 1999) has been primarily attributed to neurohumoral pathways and may involve both the RAS and the sympathetic nervous system (Zelis and Flaim, 1982; Gschwend et al., 2003). The increased vascular resistance is considered a compensatory mechanism to maintain organ perfusion by sustaining blood pressure in a failing heart. Increased vasoconstriction appears to be tightly linked to increased levels of local Ang II or constitutive upregulation of the AT1R. The fact that Ang II generation is increased and ELA-2 is responsible for Ang II generation in mesenteric resistance arteries from mice submitted to myocardial infarction, these findings provide novel information for the role played by ELA-2 upon vascular resistance in this pathological condition.

The increased parasympathetic and decreased sympathetic modulation of the heart in ELA-2KO mice may have determined chronotropic and inotropic attenuation, which may affect the cardiac output, stroke volume and left ventricular dimension. Ang II exerts direct positive chronotropic and inotropic effects (Beaulieu and Lambert, 1998; Patil et al., 2011). Studies performed in mice overexpressing ACE in the cardiac tissue demonstrated that increased Ang II generation produced a mouse phenotype with cardiac electrical abnormalities associated with a high incidence of sudden death (Xiao et al., 2004; Kasi et al., 2007). By overexpressing ACE only in the hearts of mice, Xiao et al. (2004) observed an enlargement of the atrial area without ventricular alterations, but remarkable cardiac arrhythmia and an incidence of sudden death. These authors concluded that Ang II produced by ACE does not necessarily promote ventricular enlargement or dysfunction, but can cause abnormal cardiac electrical activity. In the current study, the deletion of ELA-2 reduced both basal chrono- and inotropism, leading to a decrease in basal heart rate and stroke volume. These findings indicate that ELA-2 in cardiac tissue may play a role in maintaining basal heart rate and cardiac contractility.

Some limitations of this study should be acknowledged. First, it is not clear whether these autonomic and morphologic changes are specifically due to a reduction in local or circulating Ang II generation or due to another unknown mechanism driven by ELA-2 deficiency. Second, ACE inhibitors or angiotensin receptor antagonists were not used in the experiments. This pharmacological approach could provide more information regarding the participation of Ang II in ELA-2KO.

In summary, the present study provides direct evidence that ELA-2 has a functional role in cardiocirculatory regulation under physiological conditions in mice. The deletion of ELA-2 resulted in a decrease in basal heart rate and stroke volume, indicating that ELA-2 from the heart may play a role in maintaining the basal heart rate and cardiac contractility. These data indicate that ELA-2 may play a role in basal cardiac function and peripheral resistance by itself through cardiac autonomic modulation.

## Conclusion

These results provide the first evidence that ELA-2 modulates the sympathovagal balance in the hearts mice, as demonstrated by the reduced sympathetic modulation of the heart rate in ELA-2KO animals.

## Author contributions

Conception and design: CB, MS, MB, HS. Data collection, CB, MD, AG, RL, CP, MO, SC, PP. Analysis and interpretation, CB, MD, AG, RL, CP, MO, SC, PP. Statistical analysis, MR, CB, MD. Writing the article, CB, MD, MR. Critical revision of the article, AG, MB, JP, MR. Final approval of the article, CB, MD, AG, SB, JP, HS.

## Funding

This study was supported by grants from Fundação de Amparo à Pesquisa do Estado de São Paulo (FAPESP 2013/20549-7) and Conselho Nacional de Desenvolvimento Científico e Tecnológico (CNPq 475715/2012-8), Brazil. CB received a post-doctoral fellowship from FAPESP (2009/50548-7).

### Conflict of interest statement

The authors declare that the research was conducted in the absence of any commercial or financial relationships that could be construed as a potential conflict of interest.

## References

[B1] Albarwani S. Al-Siyabi S. Tanira M. O. (2013). Lisinopril indifferently improves heart rate variability during day and night periods in spontaneously hypertensive rats. Physiol. Res. 62, 237–245. 23489185 10.33549/physiolres.932425

[B2] Beaulieu P. Lambert C. (1998). Peptidic regulation of heart rate and interactions with the autonomic nervous system. Cardiovasc. Res. 37, 578–585. 10.1016/S0008-6363(97)00305-2 9659441

[B3] Becari C. Silva M. A. Durand M. T. Prado C. M. Oliveira E. B. Ribeiro M. S.. (2017). Elastase-2, an angiotensin II-generating enzyme, contributes to increased Ang II in resistance arteries of mice with myocardial infarction. Br. J. Pharmacol. [Epub ahead of print]. 10.1111/bph.13755 28222221 PMC5406290

[B4] Becari C. Sivieri D. O. Santos C. F. Moysés M. K. Oliveira E. B. Salgado M. C. O. (2005). Role of elastase-2 as an angiotensin II-forming enzyme in rat carotid artery. J. Cardiovasc. Pharmacol. 46, 498–504. 10.1097/01.fjc.0000177982.68563.98 16160604

[B5] Becari C. Teixeira F. R. Oliveira E. B. Salgado M. C. O. (2011). Angiotensin-converting enzyme inhibition augments the expression of rat elastase-2, an angiotensin II-forming enzyme. Am. J. Physiol. Heart Circ. Physiol. 301, H565–H570. 10.1152/ajpheart.00534.2010 21602471

[B6] Bertinieri G. di Rienzo M. Cavallazzi A. Ferrari A. U. Pedotti A. Mancia G. (1985). A new approach to analysis of the arterial baroreflex. J. Hypertens. Suppl. 3, S79–S81. 2856787

[B7] Bezerra S. M. M. S. Santos C. M. dos Moreira E. D. Krieger E. M. Michelini L. C. (2001). Chronic AT1 receptor blockade alters autonomic balance and sympathetic responses in hypertension. Hypertension 38, 569–575. 10.1161/hy09t1.095393 11566933

[B8] Brasch H. Sieroslawski L. Dominiak P. (1993). Angiotensin II increases norepinephrine release from atria by acting on angiotensin subtype 1 receptors. Hypertension 22, 699–704. 10.1161/01.HYP.22.5.699 8225530

[B9] Chen Y. Joaquim L. F. Farah V. M. Wichi R. B. Fazan R. Salgado H. C.. (2005). Cardiovascular autonomic control in mice lacking angiotensin AT1a receptors. Am. J. Physiol. Regul. Integr. Comput. Physiol. 288, R1071–R1077. 10.1152/ajpregu.00231.2004 15576667

[B10] Chompoosan C. Buranakarl C. Chaiyabutr N. Chansaisakorn W. (2014). Decreased sympathetic tone after short-term treatment with enalapril in dogs with mild chronic mitral valve disease. Res. Vet. Sci. 96, 347–354. 10.1016/j.rvsc.2014.01.006 24559801

[B11] Di Rienzo M. Parati G. Castiglioni P. Tordi R. Mancia G. Pedotti A. (2001). Baroreflex effectiveness index: an additional measure of baroreflex control of heart rate in daily life. Am. J. Physiol. Regul. Integr. Comp. Physiol. 280, R744–R751. Available online at: http://ajpregu.physiology.org/content/280/3/R744.long 11171653 10.1152/ajpregu.2001.280.3.R744

[B12] Durand M. T. Becari C. Oliveira M. de do Carmo J. M. Aguiar Silva C. A. Prado C. M.. (2014). Pyridostigmine restores cardiac autonomic balance after small myocardial infarction in mice. PLoS ONE 9:e104476. 10.1371/journal.pone.0104476 25133392 PMC4136726

[B13] Durand M. T. Becari C. Tezini G. C. S. V. Fazan R. Oliveira M. Guatimosim S.. (2015). Autonomic cardiocirculatory control in mice with reduced expression of the vesicular acetylcholine transporter. Am. J. Physiol. Heart Circ. Physiol. 309, H655–H662. 10.1152/ajpheart.00114.2015 26092977

[B14] Farquharson C. A. J. Struthers A. D. (2002). Gradual reactivation over time of vascular tissue angiotensin I to angiotensin II conversion during chronic lisinopril therapy in chronic heart failure. J. Am. Coll. Cardiol. 39, 767–775. 10.1016/S0735-1097(02)01689-3 11869839

[B15] Fazan R. Jr. de Oliveira M. da Silva V. J. D. Joaquim L. F. Montano N. Porta A.. (2005). Frequency-dependent baroreflex modulation of blood pressure and heart rate variability in conscious mice. Am. J. Physiol. Heart Circ. Physiol. 289, H1968–H1975. 10.1152/ajpheart.01224.2004 15951338

[B16] Feldstein C. A. (2014). Lowering blood pressure to prevent stroke recurrence: a systematic review of long-term randomized trials. J. Am. Soc. Hypertens. 8, 503–513. 10.1016/j.jash.2014.05.002 25064772

[B17] Gschwend S. Henning R. H. Pinto Y. M. de Zeeuw D. van Gilst W. H. Buikema H. (2003). Myogenic constriction is increased in mesenteric resistance arteries from rats with chronic heart failure: instantaneous counteraction by acute AT1 receptor blockade. Br. J. Pharmacol. 139, 1317–1325. 10.1038/sj.bjp.0705367 12890711 PMC1573962

[B18] Guzzetti S. Borroni E. Garbelli P. E. Ceriani E. Bella P. D. Montano N.. (2005a). Symbolic dynamics of heart rate variability A probe to investigate cardiac autonomic modulation. Circulation 112, 465–470. 10.1161/CIRCULATIONAHA.104.518449 16027252

[B19] Guzzetti S. Borroni E. Garbelli P. E. Ceriani E. Della Bella P. Montano N.. (2005b). Symbolic dynamics of heart rate variability: a probe to investigate cardiac autonomic modulation. Circulation 112, 465–470. 10.1161/CIRCULATIONAHA.104.518449 16027252

[B20] Hansson L. Lindholm L. H. Niskanen L. Lanke J. Hedner T. Niklason A.. (1999). Effect of angiotensin-converting-enzyme inhibition compared with conventional therapy on cardiovascular morbidity and mortality in hypertension: the Captopril Prevention Project (CAPPP) randomised trial. Lancet 353, 611–616. 10.1016/S0140-6736(98)05012-0 10030325

[B21] Huikuri H. V. Stein P. K. (2013). Heart rate variability in risk stratification of cardiac patients. Prog. Cardiovasc. Dis. 56, 153–159. 10.1016/j.pcad.2013.07.003 24215747

[B22] Jandeleit-Dahm K. A. M. Tikellis C. Reid C. M. Johnston C. I. Cooper M. E. (2005). Why blockade of the renin-angiotensin system reduces the incidence of new-onset diabetes. J. Hypertens. 23, 463–473. 10.1097/01.hjh.0000160198.05416.72 15716683

[B23] Joaquim L. F. Farah V. M. Bernatova I. Fazan R. Grubbs R. Morris M. (2004). Enhanced heart rate variability and baroreflex index after stress and cholinesterase inhibition in mice. Am. J. Physiol. Heart Circ. Physiol. 287, H251–H257. 10.1152/ajpheart.01136.2003 14988080

[B24] Jorde U. P. Ennezat P. V. Lisker J. Suryadevara V. Infeld J. Cukon S.. (2000). Maximally recommended doses of angiotensin-converting enzyme (ACE) inhibitors do not completely prevent ACE-mediated formation of angiotensin II in chronic heart failure. Circulation 101, 844–846. 10.1161/01.CIR.101.8.844 10694521

[B25] Kasi V. S. Xiao H. D. Shang L. L. Iravanian S. Langberg J. Witham E. A.. (2007). Cardiac-restricted angiotensin-converting enzyme overexpression causes conduction defects and connexin dysregulation. Am. J. Physiol. Heart Circ. Physiol. 293, H182–H192. 10.1152/ajpheart.00684.2006 17337599 PMC3160110

[B26] Kilkenny C. Browne W. Cuthill I. C. Emerson M. Altman D. G. NC3Rs Reporting Guidelines Working, Group (2010). Animal research: reporting *in vivo* experiments: the ARRIVE guidelines. Br. J. Pharmacol. 160, 1577–1579. 10.1111/j.1476-5381.2010.00872.x 20649561 PMC2936830

[B27] Lataro R. M. Silva C. A. A. Fazan R. Jr. Rossi M. A. Prado C. M. Godinho R. O.. (2013). Increase in parasympathetic tone by pyridostigmine prevents ventricular dysfunction during the onset of heart failure. Am. J. Physiol. Regul. Integr. Comp. Physiol. 305, R908–R916. 10.1152/ajpregu.00102.2013 23948774

[B28] Laude D. Baudrie V. Elghozi J.-L. (2008). Applicability of recent methods used to estimate spontaneous baroreflex sensitivity to resting mice. Am. J. Physiol. Regul. Integr. Comp. Physiol. 294, R142–R150. 10.1152/ajpregu.00319.2007 17989145

[B29] Lindholm L. H. Ibsen H. Borch-Johnsen K. Olsen M. H. Wachtell K. Dahlöf B.. (2002). Risk of new-onset diabetes in the Losartan Intervention For Endpoint reduction in hypertension study. J. Hypertens. 20, 1879–1886. 10.1097/00004872-200209000-00035 12195132

[B30] Mansier P. Médigue C. Charlotte N. Vermeiren C. Coraboeuf E. Deroubai E.. (1996). Decreased heart rate variability in transgenic mice overexpressing atrial beta 1-adrenoceptors. Am. J. Physiol. 271, H1465–1472. 8897941 10.1152/ajpheart.1996.271.4.H1465

[B31] McGrath J. C. Drummond G. B. McLachlan E. M. Kilkenny C. Wainwright C. L. (2010). Guidelines for reporting experiments involving animals: the ARRIVE guidelines. Br. J. Pharmacol. 160, 1573–1576. 10.1111/j.1476-5381.2010.00873.x 20649560 PMC2936829

[B32] McGrath J. C. McLachlan E. M. Zeller R. (2015). Transparency in research involving animals: the basel declaration and new principles for reporting research in BJP manuscripts. Br. J. Pharmacol. 172, 2427–2432. 10.1111/bph.12956 25899710 PMC4409896

[B33] Menezes A. da S. Moreira H. G. Daher M. T. (2004). Analysis of heart rate variability in hypertensive patients before and after treatment with angiotensin II-converting enzyme inhibitors. Arq. Bras. Cardiol. 83, 169–172. 10.1590/S0066-782X2004001400008 15322659

[B34] Patil J. Stucki S. Nussberger J. Schaffner T. Gygax S. Bohlender J.. (2011). Angiotensinergic and noradrenergic neurons in the rat and human heart. Regul. Pept. 167, 31–41. 10.1016/j.regpep.2010.11.011 21145919

[B35] Porta A. Tobaldini E. Guzzetti S. Furlan R. Montano N. Gnecchi-Ruscone T. (2007). Assessment of cardiac autonomic modulation during graded head-up tilt by symbolic analysis of heart rate variability. Am. J. Physiol. Heart Circ. Physiol. 293, H702–H708. 10.1152/ajpheart.00006.2007 17308016

[B36] Reid I. A. (1992). Interactions between ANG II, sympathetic nervous system, and baroreceptor reflexes in regulation of blood pressure. Am. J. Physiol. 262, E763–E778. 1616014 10.1152/ajpendo.1992.262.6.E763

[B37] Sabino J. P. J. da Silva C. A. A. de Melo R. F. Fazan R. J. Salgado H. C. (2013). The treatment with pyridostigmine improves the cardiocirculatory function in rats with chronic heart failure. Auton. Neurosci. Basic Clin. 173, 58–64. 10.1016/j.autneu.2012.11.007 23218833

[B38] Santos C. F. Caprio M. A. V. Oliveira E. B. Salgado M. C. O. Schippers D. N. Munzenmaier D. H.. (2003). Functional role, cellular source, and tissue distribution of rat elastase-2, an angiotensin II-forming enzyme. Am. J. Physiol. Heart Circ. Physiol. 285, H775–H783. 10.1152/ajpheart.00818.2002 12714330

[B39] Santos C. F. Oliveira E. B. Salgado M. C. O. Greene A. S. (2002). Molecular cloning and sequencing of the cDNA for rat mesenteric arterial bed elastase-2, an angiotensin II-forming enzyme. J. Cardiovasc. Pharmacol. 39, 628–635. 10.1097/00005344-200205000-00002 11973405

[B40] Schrier R. W. Abraham W. T. (1999). Hormones and hemodynamics in heart failure. N.Engl. J. Med. 341, 577–585. 10.1056/NEJM199908193410806 10451464

[B41] Stein P. K. (2013). Challenges of heart rate variability research in the ICU. Crit. Care Med. 41, 666–667. 10.1097/CCM.0b013e318270e6f0 23353948

[B42] Takata Y. Arai T. Suzuki S. Kurihara J. Uezono T. Okubo Y.. (2004). Captopril enhances cardiac vagal but not sympathetic neurotransmission in pithed rats. J. Pharmacol. Sci. 95, 390–393. 10.1254/jphs.SCJ04003X 15272216

[B43] Te Riet L. van Esch J. H. M. Roks A. J. M. van den Meiracker A. H. Danser A. H. J. (2015). Hypertension: renin-angiotensin-aldosterone system alterations. Circ. Res. 116, 960–975. 10.1161/CIRCRESAHA.116.303587 25767283

[B44] Thayer J. F. Yamamoto S. S. Brosschot J. F. (2010). The relationship of autonomic imbalance, heart rate variability and cardiovascular disease risk factors. Int. J. Cardiol. 141, 122–131. 10.1016/j.ijcard.2009.09.543 19910061

[B45] Thireau J. Zhang B. L. Poisson D. Babuty D. (2008). Heart rate variability in mice: a theoretical and practical guide. Exp. Physiol. 93, 83–94. 10.1113/expphysiol.2007.040733 17911354

[B46] Tobaldini E. Porta A. Wei S.-G. Zhang Z.-H. Francis J. Casali K. R.. (2009). Symbolic analysis detects alterations of cardiac autonomic modulation in congestive heart failure rats. Auton. Neurosci. Basic Clin. 150, 21–26. 10.1016/j.autneu.2009.03.009 19403339 PMC2931340

[B47] Wickman K. Nemec J. Gendler S. J. Clapham D. E. (1998). Abnormal heart rate regulation in GIRK4 knockout mice. Neuron 20, 103–114. 10.1016/S0896-6273(00)80438-9 9459446

[B48] Wu L. Jiang Z. Li C. Shu M. (2014). Prediction of heart rate variability on cardiac sudden death in heart failure patients: a systematic review. Int. J. Cardiol. 174, 857–860. 10.1016/j.ijcard.2014.04.176 24804906 PMC4318838

[B49] Xiao H. D. Fuchs S. Campbell D. J. Lewis W. Dudley S. C. Kasi V. S.. (2004). Mice with cardiac-restricted Angiotensin-Converting Enzyme (ACE) Have atrial enlargement, cardiac arrhythmia, and sudden death. Am. J. Pathol. 165, 1019–1032. 10.1016/S0002-9440(10)63363-9 15331425 PMC1618615

[B50] Yusuf S. Sleight P. Pogue J. Bosch J. Davies R. Dagenais G. (2000). Effects of an angiotensin-converting-enzyme inhibitor, ramipril, on cardiovascular events in high-risk patients. Heart outcomes prevention evaluation study investigators. N. Engl. J. Med. 342, 145–153. 10.1056/NEJM200001203420301 10639539

[B51] Zelis R. Flaim S. F. (1982). Alterations in vasomotor tone in congestive heart failure. Prog. Cardiovasc. Dis. 24, 437–459. 10.1016/0033-0620(82)90012-3 6805039

