# Supplementary material for: Elastase-2, a Tissue Alternative Pathway for Angiotensin II Generation, Plays a Role in Circulatory Sympathovagal Balance in Mice
Source: Front Physiol. 2017 Mar 23;8:170. doi: 10.3389/fphys.2017.00170 (PMC5363176; doi:10.3389/fphys.2017.00170)
Supplement: Supplementary file 1 [file DataSheet1.DOCX]

**Elastase-2, a tissue alternative pathway for angiotensin II generation, plays a role in circulatory sympathovagal balance in mice**

Christiane Becari^1,3,6*^, Marina T. Durand^1,5*^, Alessander O. Guimaraes^7,10^, Renata M. Lataro^1^, Cibele M. Prado^2^, Mauro de Oliveira^1^, Sarai C. O. Candido^1^_,_ Paloma Pais^1^, Mauricio S. Ribeiro^4^, Michael Bader^7,8,9^, Joao B. Pesquero^10^, Maria Cristina O. Salgado^3^, Helio C. Salgado^1^

Departments of ^1^Physiology, ^2^Pathology and ^3^Pharmacology, ^4^Surgery and Anatomy, Ribeirao Preto Medical School, University of Sao Paulo, Ribeirao Preto-SP, Brazil. ^5^Department of Medicine, University of Ribeirão Preto, Ribeirão Preto-SP, Brazil. ^6^Department of Cardiovascular Diseases, Mayo Clinic, Rochester, MN, USA. ^7^Max Delbrück Center for Molecular Medicine, ^8^Berlin Institute of Health (BIH), Charité - University Medicine Berlin, and ^9^DZHK (German Center for Cardiovascular Research), partner site Berlin, Berlin, Germany. ^10^Department of Biophysics, Federal University of São Paulo, Brazil.

**Corresponding author:** Helio C. Salgado, M.D. Ph.D

Department of Physiology, Ribeirao Preto Medical School,

Av. Bandeirantes, 3900

14049-900, Ribeirão Preto, SP, Brazil

Phone: 55 (16) 3315 3201

E-mail: hcsalgado@fmrp.usp.br

* These authors contributed equally to this work.

**Short title:** Cardiovascular profile of Elastase-2 knockout mice

**SUPPLEMENTARY DATA**

**Production of the Cela2aCKO-2B9 ES cell line and the B6;129P2-Cela2aTm1Bdr mouse strain**

Conditional knockout mice for the chymotrypsin-like elastase family member 2A ([Cela2a](http://www.ncbi.nlm.nih.gov/sites/entrez?db=gene&amp;cmd=Retrieve&amp;dopt=Graphics&amp;list_uids=13706)) gene, which is located at position qE1 in chromosome 4, were generated using the vector [PRPGS00048_A_H09](http://www.sanger.ac.uk/htgt/plate/view?plate_name=PRPGS00048_A) (KOMP Repository at UC Davis, CA, USA). When homologously recombined, this vector leads to a modified Cela2a allele containing a loxP site in intron 5 and the [L1L2_Bact_P](http://www.sanger.ac.uk/htgt/cassettes#L1L2_Bact_P) targeting transgenic cassette in intron 2 (Figure 1). This cassette harbors the mouse En2 gene intron/exon splice acceptor between FRT sites followed by a frame independent *beta-galactosidas*e transgene and the neomycin phosphotransferase gene under transcriptional control of the mouse beta actin minimal promoter. Therefore, the resulting mouse strain will carry a defective Cela2a allele and can be used to assess the temporal and spatial transcription of the Cela2a gene by monitoring Beta-galactosidase activity, in addition to being suitable for evaluating the effect of Cela2a gene conditional disruption by crossing this strain with specific Cre and Flp recombinase transgenic strains.

We first obtained the CelaCKO-2B9 embryonic stem cells (ES cells) by electroporating the targeting plasmid [PRPGS00048_A_H09](http://www.sanger.ac.uk/htgt/plate/view?plate_name=PRPGS00048_A) into the wild-type 129/OlaHsd mouse ES cells and screening 192 of the resulting G418 resistant ES cell clones. Only clone 2B9 had a recombination event with the targeting plasmid that correctly modified the Cela2a locus, as evidenced by PCR and long-range PCRs (Figure 2).

The B6;129P2-Cela2aTm1Bdr conditional knockout mouse strain was then generated by C57Bl/6 blastocyst micro-injection of the CelaCKO-2B9 ES cells and transfer to foster mothers. We obtained 2 male chimeric mice, both containing C57Bl/6 blastocyst and CelaCKO-2B9 ES cell derived cells, which were able to successfully transmit the modified Cela2aTm1Bdr allele to offspring. These resulting F1 generation heterozygous males and females were subsequently used to obtain homozygous mice for the Cela2aTm1Bdr allele, which are viable and did not have any striking phenotypic difference from wild-type litter mates.

**Confirming the Cela2a gene disruption by lack of Cela2a expression in the spleens of B6;129P2- Cela2aTm1Bdr homozygous animals but not of wild type.**

We obtained total RNA from spleens and thymus of a 5 week old B6;129P2-Cela2aTm1Bdr homozygous male and a wild-type brother, and used it to analyze the Cela2a expression by two independent reverse transcription polymerase chain reactions (RT-PCRs). RT-PCR1 was performed using primers pElaE2f and pElaWTr2 to amplify a 84 base pair fragment, and RT-PCR2 was performed using primers ElaF and ElaE6r to amplify a 151 base pair amplicon. The good quality of the cDNA produced from the total RNAs was assured by performing a RT-PCR to detect the mouse Tbp expression (Ref Seq. [NP_038712.3](http://www.ncbi.nlm.nih.gov/protein/NP_038712.3)), which yielded the expected 117 bp fragments (Figure 3a) as described in the methods. We detected only the expected amplicons for the RT-PCRs 1 and 2 in the wild-type spleen sample, confirming the disruption of the Cela2a gene expression (Figure 3 b and c).

# Methods:

The care and handling of all mice used for these experiments were performed according to the guidelines for the humane use of laboratory animals by the Max-Delbrück-Center for Molecular Medicine animal facility and approved by a local ethical committee.

# Mouse CelaCKO-2B9 embryonic stem cell production.

The 129/OlaHsd mouse ES cells were seeded on mitotically inactivated (by exposure for 2.5 hours to 10 ng/ml of mitomycin C (Sigma)) C57Bl/6 **wild-type** mouse embryonic fibroblasts (MEFs) and cultured in ES cell medium consisting of: Dulbecco’s modified Eagle’s medium (DMEM), 15% FCS, and 1,000 U/ml of LIF (ESGRO, Life Technologies, Gaithersburg, MD, USA) with supplements. The cells were transfected by electroporation (single pulse of 0.5 mF, 240 mV) with the targeting vector [PRPGS00048_A_H09](http://www.sanger.ac.uk/htgt/plate/view?plate_name=PRPGS00048_A) (KOMP Repository at UC Davis, CA, USA), previously replicated in and purified from a *E. coli* DH5α strain, linearized with the restriction enzyme NsiI (New England Biolabs) and agarose gel purified using the QiaEX Gel purification Kit (Qiagen). After approximately 10 days of treatment with G418 (200 mg/ml, Gibco), 192 neomycin-resistant clones were selected and individually expanded and split into two samples to obtain genomic DNA for analysis and frozen for future use.

Genomic DNA (gDNA) from the 192 clones, obtained by ethanol precipitation after proteinase K digestion for 4 hours in lysis buffer (100 micrograms of proteinase K in 1 ml of 100 mM Tris pH 8.0, 5 mM EDTA pH 8, 0.2% SDS, and 200 mM NaCl), was first analyzed by PCR 1, with primers ELAlong5 (5' CATGGGAGCCTTCAACAATC 3') and ELAlong3 (5' CGCCATAGTGACTGGATATG 3') that amplify a 312 bp fragment only from clones in which random genomic integration of the vector occurred (Figure 2A).

The gDNA of PCR1 negative clones were then analyzed by PCR2, with the primers pELAloxf2 (5' AGGCACATTGAGATGGCGC 3´) and pELAloxr2 (5' TTCTTGAACTGATGGCGAGC 3') that amplify a 93 bp fragment only from clones in which the loxP site in the Cela2a intron 5 is present (Figure 2B). The gDNA from PCR 2 positive clones was subjected to analysis by two long range PCRs: LRPCR1, which is amplifiedy with primers Ela5oF1 (5'CTGAGAAGAGTGCCACCTGC3') and pmEn2ieR2 (5'GACCTTGGGACCACCTCATCAG 3') yielding a 4880pb fragment; and LRPCR2, which produces a 8425 bp amplicon with primers ElaMarF1 (5' CCCAACCTGCCATCACGAG 3') and pELApr3r (5'GCTCTTTCAGGAGGAGATTG 3') (Figures 2C, 2D). These LRPCRs were performed using the Advantage® 2 PCR Kit (Clontech) as suggested by the manufacturer with the following temperature protocol: 95°C for 3 minutes followed by 40 cycles of 95°C for 15 seconds, 60°C for 15 seconds and 68°C for 5 minutes for LRPCR1 and 9 minutes for LRPCR2.

# B6;129P2-Cela2aTm1Bdr conditional knockout mouse strain production.

Only the clone CelaCKO-2B9 yielded the expected LRPCR1 and 2 amplicons and was then thawed, seeded on mitotically inactivated C57Bl/6 **wild-type** MEFs and cultured as described previously.

The CelaCKO-2B9 ES cells were grown to subconfluency in 25 cm^2^ flasks, trypsinized for 3-4 minutes, washed once in PBS, resuspended in 5 ml of ES cell medium without LIF, replated and maintained in culture to allow the MEFs to adhere to the plate. After 30 minutes, the medium containing the yet unattached ES cells was collected, the cells pelleted by centrifugation and resuspended to 2x 105 cells/ ml in cold ES cell medium without LIF and stored on ice (for up to several hours) to prevent clumping.

Blastocysts were flushed from pregnant C57Bl/6 females and collected into a CO_2_- independent medium (Gibco-BRL) containing 10% FBS. Blastocysts were expanded for 1-2 hours in ES cell medium in a 37°C/6% CO2 incubator, transferred to a hanging drop chamber, and cooled to 4°C. The collected ES cells were added to the hanging drops, and the blastocysts were injected with enough cells (20 or more) to fill the blastocoele. Injected blastocysts were then transferred to pseudopregnant recipient females (10-15 blastocysts/uterine horn).

Two male animals that were born showed the characteristic patches of cream colored fur, indicating that they were chimeric animals with cells derived from the C57Bl/6 blastocyst and 129/Ola ES cells because the latter carries the recessive pinkeye (p) and chinchilla (cch) mutations. These 2 males were further crossed to C57Bl/6 females and germ line transmission of the modified Cela2aTm1Bdr allele was confirmed by tail genomic DNA (gDNA) genotyping PCRs of the offspring (additional information 1).

# Confirming Cela2a gene disruption by lack of Cela2a expression in spleen of B6;129P2- Cela2aTm1Bdr homozygous animals

Spleens and thymus of a 5 week old B6;129P2-Cela2aTm1Bdr homozygous male and his **wild-type** brother were collected and immediately frozen in liquid nitrogen after the animals were sacrificed by decapitation. Approximately 2 mm of the tail tip of each animal was also collected and processed to confirm the genotypes.

The spleen and thymus samples were homogenized in TRIzol reagent (Invitrogen) and total RNA was obtained as instructed by the manufacturer and resuspended in MilliQ (Millipore) purified water. The RNA concentration and purity were measured with the NanoDrop 1000 (Thermo Scientific) instrument and their integrity was confirmed by visualization of the rRNAs bands after electrophoresis of 500 ng of total RNA/per sample in a 1% agarose Gel stained with ethidium bromide under UV illumination (data not shown). We then obtained total cDNA from 4 micrograms of purified total RNAs from each sample using M-MLV reverse transcriptase (Invitrogen) and random hexamers as instructed in the product manual. For each RT subsequent PCR, we used 5 µl of a 1/50 diluted total cDNA as template in a 30 µl final volume reaction and 2 units of recombinant Taq DNA polymerase (Invitrogen) in the recommended product buffer supplemented with 1 microliter of the following: 50 mM MgCl_2_, 5 mM dNTPs, each primer at 5 mM and genomic DNA (at approximately 100 ng/microliter) per reaction. The temperature protocol used was as follows: 95°C for 3 minutes followed by 40 cycles of 95°C for 15 seconds, 60°C for 15 seconds and 72°C for 30 seconds.

The following primers were used in the PCR portion of the above described RT-PCRs: pElaE2f (5´GAGGATGATGTGAGCAGGGT3´); pElaWTr2 (5´GGAAAGGACCTGCAGGGAG 3´); ElaF (5´AGAAACTATGTCTGCTATGTCAC 3´); ElaE6r(5´CCATGCTGGACTTCACAGAG 3´); mrTBPf (5´ CCCTATCACTCCTGCCACACC 3´); and hmrTBPr (5´ CGAAGTGCAATGGTCTTTAGGTC 3´)

**SUPPLEMENTARY FIGURES**


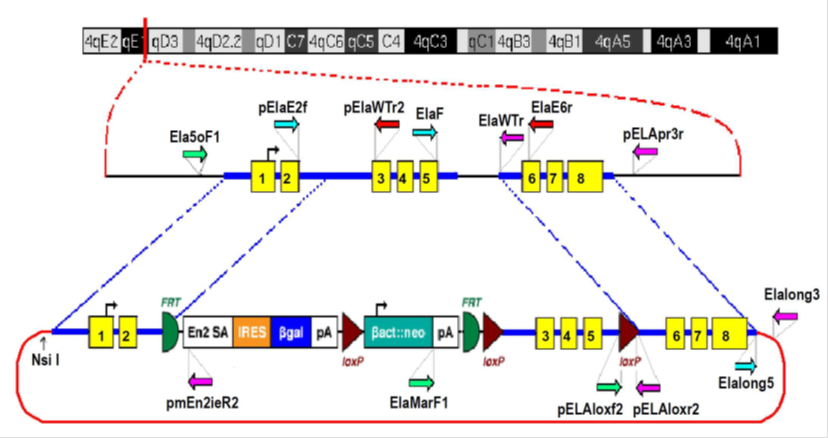


**Supplementary Figure 1:** **Representation of the correct recombination events between the mouse Cela2a gene and the targeting plasmid PRPGS00048_A_H09 generating the Cela2aTm1Bdr mutated allele.** The position of the Cela2a locus on a schematic representation of the mouse chromosome 4 (chr4) is shown as a red vertical line. The Cela2a exons are represented as numbered yellow boxes in both chromosome and targeting plasmid schemes. The Cela2a locus genomic sequence in the plasmid is represented as blue thick lines and the correct recombination event region connected by dotted blue lines. The unique plasmid sequence is represented as a red line, the reading frame-independent lacZ gene trap cassette is represented by a white-orange-blue box and the G418 drug selection cassette is represented by a light blue-white box between indicating the FRT (green half circle) and loxP sites (brown triangles). The following plasmid cassette elements are also represented: mouse En2 intron/exon splice acceptor (En2 SA), encephalo myeloid virus internal ribosome entry sequence (IRES), lacZ gene (βgal), simian virus 40 polyadenylation site (pA), human β-actin promoter and neomycin phosphotransferase (β act:neo). Forward and reverse primers are indicated as green, light blue, pink or red arrows and are described in the text.

**
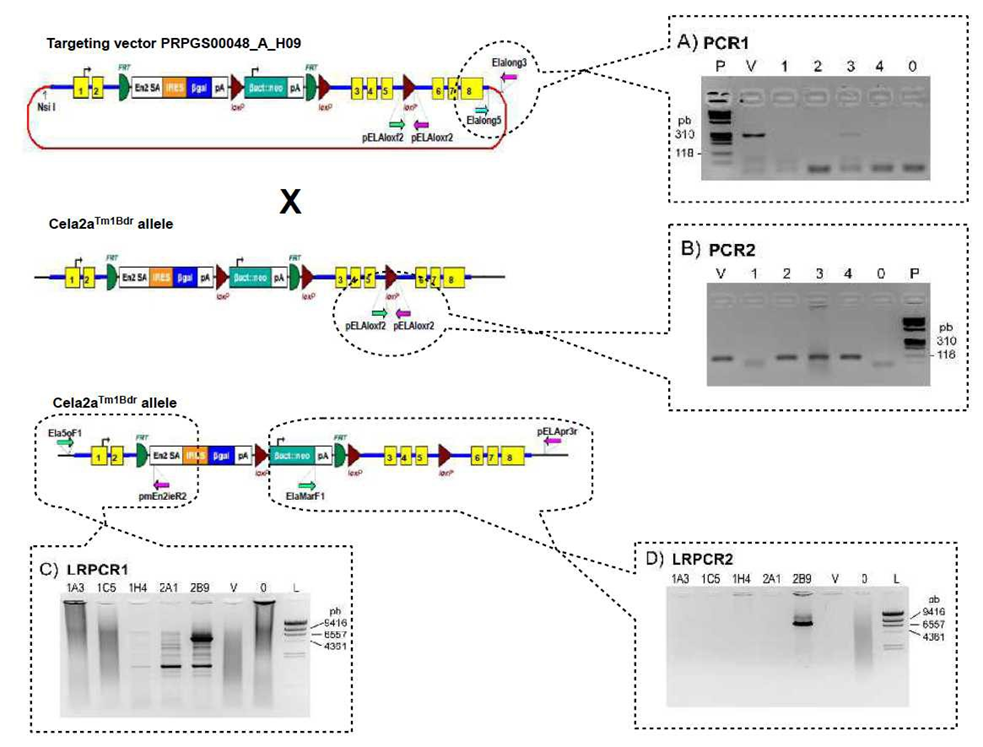
**

**Supplementary Figure 2:** PCR analysis of some G418 resistant 129/OlaHsd ES cell colonies after targeting plasmid PRPGS00048_A_H09 electroporation and G418 selection. Schematic representation of the wild-type Cela2a allele, the targeting plasmid and the Cela2aTm1Bdr allele showing the positions of the screening PCRs primers indicated (colored arrows) and representative negative pictures of 2% agarose gels stained with ethidium bromide and visualized under UV light after electrophoresis of the screening PCRs indicated. **A)** Representative PCR1 screening of 4 samples (1 to 4). **B)** Representative PCR2 screening of 4 PCR1 negative samples (1 to 4). **C)** Long range PCR1 performed in the indicated samples. **D)** Long range PCR2 performed in the indicated samples. In all gel pictures, the non-template control is indicated as sample 0, and V are the positive control PCRs using the targeting plasmid as a template (approximately 0,001 ng/reaction). The ΦX174 RF DNA/*Hae* III fragments (P) and λ DNA/*Hin*d III fragments (L) from Invitrogen were used, and band sizes are indicated in base pairs (bp).


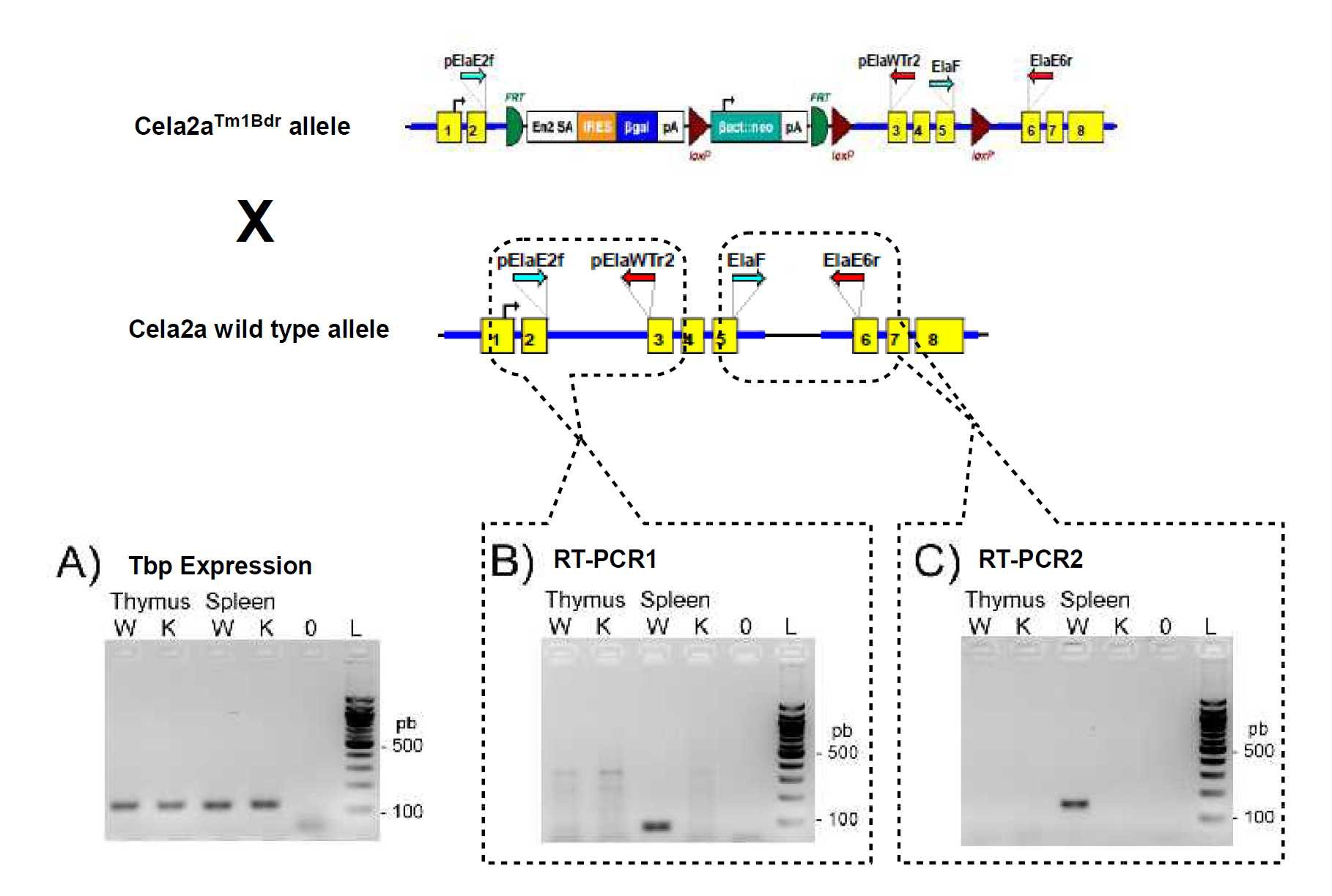
 **Supplementary Figure 3: RT-PCR analysis of B6;129P2-Cela2aTm1Bdr homozygous (K) and litter mate wild-type control mice (W) thymus and spleen samples.** Schematic representation of the **wild-type** and Cela2aTm1Bdr allele indicating the primers used in the RT-PCRs (colored arrows). Negative pictures of 2% agarose gels stained with ethidium bromide and visualized under UV light after electrophoresis of the Tbp detection RT-PCR (A), RT-PCR1 (B) and RT-PCR2 (C), of samples from a **wild-type** male (W), a litter mate 129P2-Cela2aTm1Bdr homozygous male (K) or no template (0). The GeneRulertm100bp DNA ladder (Fermentas) was used as a DNA size reference (L) and some band sizes are indicated.
